# Multi-Layer Approach for the Detection of Selective Forwarding Attacks

**DOI:** 10.3390/s151129332

**Published:** 2015-11-19

**Authors:** Naser Alajmi, Khaled Elleithy

**Affiliations:** Computer Science and Engineering Department, University of Bridgeport, Bridgeport, CT 06604, USA

**Keywords:** wireless sensor networks, selective forwarding attacks

## Abstract

Security breaches are a major threat in wireless sensor networks (WSNs). WSNs are increasingly used due to their broad range of important applications in both military and civilian domains. WSNs are prone to several types of security attacks. Sensor nodes have limited capacities and are often deployed in dangerous locations; therefore, they are vulnerable to different types of attacks, including wormhole, sinkhole, and selective forwarding attacks. Security attacks are classified as data traffic and routing attacks. These security attacks could affect the most significant applications of WSNs, namely, military surveillance, traffic monitoring, and healthcare. Therefore, there are different approaches to detecting security attacks on the network layer in WSNs. Reliability, energy efficiency, and scalability are strong constraints on sensor nodes that affect the security of WSNs. Because sensor nodes have limited capabilities in most of these areas, selective forwarding attacks cannot be easily detected in networks. In this paper, we propose an approach to selective forwarding detection (SFD). The approach has three layers: MAC pool IDs, rule-based processing, and anomaly detection. It maintains the safety of data transmission between a source node and base station while detecting selective forwarding attacks. Furthermore, the approach is reliable, energy efficient, and scalable.

## 1. Introduction

A sensor node is a small, lightweight sensing device. It is composed of a constrained processing unit and small amount of memory for its small operating system. Additionally, a sensor node includes a limited-range transceiver and a battery unit [1]; a mobile node also includes a mobility subsystem. WSNs often manage thousands of sensor nodes. In fact, these sensor nodes communicate with a huge number of small nodes via radio links. WSNs applications distinguish between applications based on the type of data that must be collected in the network [2]. Sensor nodes in a network gather data that are necessary to include in a smart network environment. These environments include homes, transportation systems, military installations, healthcare systems, and buildings. WSNs make it technologically possible to reorganize information and communication technology. The study of WSNs is a significant topic in computer science and engineering. It has an economic impact and affects industry.

In WSNs, sensor nodes transfer packets from the source to the base station. Because a sensor node is a limited-transmission device, it uses a multi-hop method to send packets to the base station [3]. While the communication between sensor nodes in WSNs is accomplished wirelessly by radio, adversaries can use many types of attacks. Eavesdropping, compromising nodes, interrupting or modifying packets, and injecting malicious packets compromise privacy, and denial of service attacks are threats to the security of WSNs [4]. Attackers compromise the internal sensor nodes from which they launch attacks, which are difficult to detect. A selective forwarding attack is the one of these attacks. It is an attack where a node sends some of messages to other nodes or base stations whilst dropping the sensitive information [5].

In a selective forwarding attack, malicious nodes attempt to stop the packets in a network by rejecting message forwarding. It is not easy to detect this type of attack due to the unreliability of communications. According to Karlof and Wagner [6], selective forwarding attacks can impact some routing protocols, such as TinyOS beaconing, DSR, and PSFQ. During the launch of a selective forwarding attack, a compromised node has notable consequences. A compromised node selectively drops packets. Malicious nodes work in the same manner as other nodes in the network field, but these malicious nodes try to find the important messages and drop them before sending the whole packets to the next nodes. The attackers make the sensor network rely on the redundancy forwarding by using broadcast for data to spread in the network. Based on researchers’ results, limited power and low memory are obstacles that make conventional security measures inappropriate for WSNs [7]. One of the obstacles in WSNs is also energy consumption, so data transmission between sensor nodes is the major source of energy consumption and it is a serious challenge to design an energy efficient routing scheme for extending a network’s lifetime [8].

## 2. Related Works

Yu and Xiao [9,10] described a Lightweight Security Scheme (LWSS) as an approach that can be used to detect a selective forwarding attack in the sensor network field. LWSS uses a multi-hop acknowledgment to launch alarms by obtaining responses from the nodes that are located in the middle of a path. This approach has as a target the detection of network attackers. The target is to send an alarm that indicates a selective forwarding attack when a malicious node is discovered. The authors used two detection processes in the scheme, namely a downstream process and an upstream process. Sending an acknowledgement packet and alert packet would drain energy during the detection process. In this approach, a node is randomly selected as the checkpoint that sends a message acknowledging the detection of an adversary. Among the drawbacks of LWSS we may list the following:

- Resending the packet by using another route causes energy consumption and delays during the detection.
- Transmission of the acknowledgement packet and one-way key packet also cause energy consumption.
- The scheme lacks scalability.
- The scheme spends much effort on detecting the attack thus it lacks efficiency.
- The LWSS scheme could not detect the attacks under some certain conditions.
- Sending the acknowledgment causes wasted energy.
- There is no commitment to reliability if the packet is dropped.

Hai and Huh [11] described an approach to detect selective forwarding attacks. The approach is called Lightweight Detection (LWD). It consists of a lightweight mechanism where each sensor node is provided with a detection module that is built on top of an application layer. A sensor node sets its routing rules and uses information about its two-hop neighborhood to generate an alert packet. Hai and Huh suggested two routing rules to improve the monitoring system. The first rule is to determine whether the destination node forwards the packet along the path to the sink. The second rule is that the monitoring node waits and detects a packet that had been forwarded along the path to the sink. Some of the drawbacks of LWD are:

- The network has a static topology. Therefore, the LWD scheme will not detect the attack if there is a change in the type of topology.
- There is no guarantee of reliability.
- The detection scheme is not work if a node is compromised.

Deng *et al.* [12] proposed Secure Data Transmission (SDT) for detecting a selective forwarding attack. They used watermark technology to detect malicious nodes. Prior to employing a watermark-based technique, they used a trust value to find a source path for message forwarding. When the network is initialized, all of the nodes are assigned the same trust value. Deng *et al.* used a watermark-based technique to calculate the amount of packet loss. The base station compares the extracted watermark to the original watermark to detect a selective forwarding attack. Among the drawbacks of SDT Drawbacks one can mention:

- There is no data resend method if the packet is dropped.
- The SDT scheme cannot detect a malicious node if more than two.
- The scheme is not convenient for sensor-caused malicious nodes and the BS cannot compromise.

Chanatip *et al.* [13] proposed Received Single Strength Indicator—Extra Monitor (RSSI-EM). They used extra monitoring (EM) to eavesdrop and monitor all of the traffic when data were transferred between nodes. The value of an RSSI is that four EM nodes can be arranged to establish the positions of all of the sensor nodes, with the base station located at (0, 0). They assumed that the attackers could capture and damage the nodes. Therefore, all of the sensor nodes must protect themselves or be made from tamper-resistant hardware. The RSSI-EM drawbacks are:

- The topology is static thus any change will affect the efficiency of the scheme.
- The scheme accuracy is low.

## 3. Problem Identification

**Figure 1:**
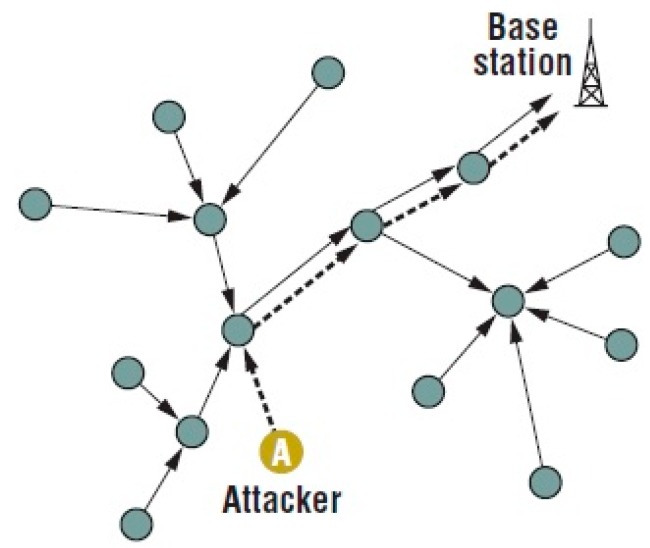
Selective Forwarding Attacks-Redrawn [15].

A selective forwarding attack is difficult to detect in a network. The adversary installs a malicious node in the network area, which drops packets. Once the malicious node is present in the network, it organizes routing loops that attract or refuse network traffic. Additionally, malicious nodes can perform some activities that impact the network. These activities include extending or shortening source routes, generating false messages, and attempting to drop significant messages (Figure 1). Packet drops are common due to environmental conditions, but it is also possible that an attacker can simply drop a packet purposefully [14]. Packets that are dropped selectively sometimes come from one node or a group of nodes. The malicious node refuses to forward the packets. In addition, the base station does not receive the entire message. There is a need for a new paradigm for detecting selective forwarding attacks that can increase the detection rate while consuming less energy.

## 4. Proposed System

Sensor networks are vulnerable to many types of security attack. A malicious node tries to create blocks that occur while messages are being transferred between sensor nodes in the network by, for instance, forwarding a message along another path, generating an inaccurate network route, and delaying the transfer of packets between nodes. With a limited radio communication range, wireless sensor nodes communicate with each other by a multi-hop path [16]. In a sensor network area, data are sent to the base station through routers. An attacker compromises the nodes by attacking the network resources. Selective forwarding attacks destroy the packets transmitted between the source and base station. For this purpose, a malicious node refuses to transfer the whole packet, attempting to drop considerable data and therefore, the whole packet is not transferred to the base station. Furthermore, physical attacks frequently occur in WSNs because they are easy for adversaries to execute. Selective forwarding attacks can seriously impact the data collection of WSNs and data will be lost with compromised sensor nodes [17]. Selective forwarding detection (SFD) discovers a secure route for data to be sent from one node to other nodes. In this section of the paper, we introduce the assumptions and a multi-layer approach for detecting selective forwarding attacks.

### 4.1. Assumptions

To detect selective forwarding attacks within certain applications we must make some assumptions. We assume that all nodes have the same specifications. All nodes in the network have the same energy at the starting point and maximum energy. As well as, we assume that nodes are uniformly distributed in network in a random manner. Malicious nodes should not drop any packets before launching a selective forwarding attack, and an adversary cannot attack nodes during their deployment. Nodes can send data to a base station. Received Signal Strength Indicator-RSSI is the mechanism to measure the distance between the base station and a node.

### 4.2. Selective Forwarding Detection (SFD) Using Multi-Layers

**Figure 2:**
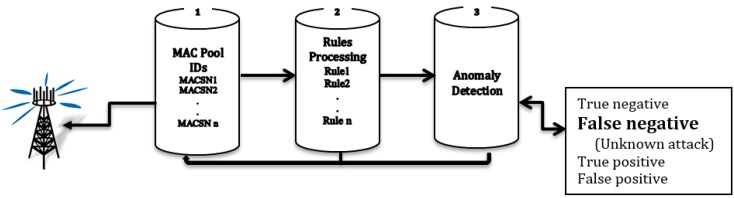
Multi-Layers in Rules-Based IDS.

**Figure 3:**
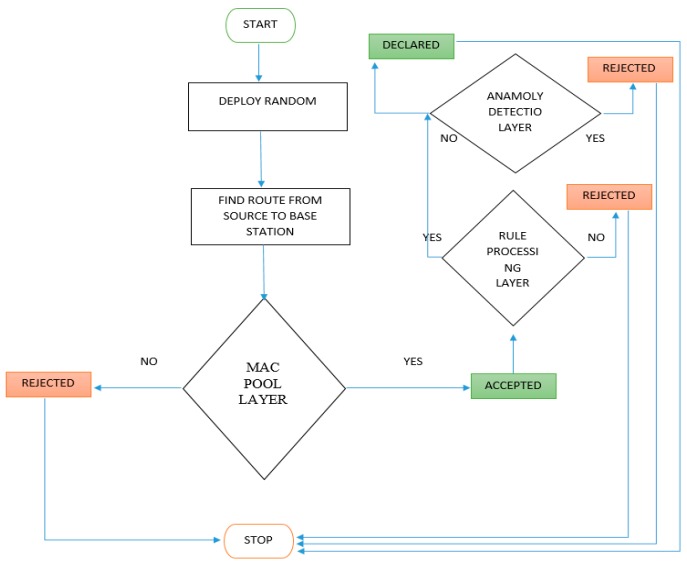
Selective Forwarding Attack Detection Flowchart.

Rule-based IDS, also known as signature-based IDS, is one of the mechanisms for protecting a network from security threats. The network layer in WSNs is threatened with many types of attacks, including wormhole and sinkhole attacks. Our proposal focuses on the selective forwarding attack. We design a multi-layer approach to detection that includes the three security layers shown in Figure 2. Figure 3 shows the details of the algorithm. The first layer is a pool of MAC IDs. In this layer, the important information is filtered and stored. The information includes message fields (e.g., packet, destination, and source IDs) that are useful for rule-based processing. The second layer is the rule-based processing layer. In this layer, there are some rules that must be applied to the stored data. Incoming traffic is either accepted or rejected. In addition, no rules are applied to a message that fails. The third layer is the anomaly detection layer, which detects the false negative anomalies that comprise unknown attacks. The second layer (rule-based processing) and the third layer (anomaly detection-based IDS) can identify and control selective forwarding attacks in all phases. The three layers are supported by three algorithms. These algorithms are used to resolve the attacks on the network. The detection approach saves energy by using little time and memory. It chooses a secure route along which to transfer data between the source and base station. Furthermore, the SFD approach will be reliable, energy efficient, and scalable. All of these factors are important for sensor node networks. Additionally, this approach has a high accuracy rate. We compared our approach with other approaches and found SFD has a 98.3% accuracy rate so it is higher than others.

### 4.3. Selective Forwarding Detection (SFD) Algorithms

#### 4.3.1. MAC Pool of IDs Layer

The first layer consists of a pool of MAC IDs that filters and matches the traffic. Each traffic packet is monitored. The packet is matched to identify malicious activity using message fields (e.g., the packet, destination, and source IDs). It checks whether a node is legitimate or malicious. If a node is assigned a value of zero, it drops a packet and is considered malicious. Otherwise, it is accepted as a legitimate node. In our study, we analyze the malicious nodes that are detected in the first step using an algorithm based on the pool of MAC IDs as shown in Algorithm 1.

**Algorithm 1.** MAC Pool of IDs Layer

1.   **Input** = (MP: Mac Pool)
2.   **Network parameter** = (SN: sensor node, RT: route, TSN: Total sensor node)
3.   For (SN = 0; SN <= TSN; SN++)
4.     Set SN = SN + 1
5.    If SN ∈ MP then
6.     Set SN = 0 // the node is declared as malicious node not allowed for communication.
7.      Rejected
8.      Dropped
9.    Else if SN = 1 // Node is declared as a legitimate node and allowed for communication
10.      Accept
11.      Store
12.     Set SN = RT
13.      SN → RP
14.    End if
15.    End else
16.   End for

#### 4.3.2. Rules Processing Layer

The second layer involves rule-based processing. It is the middle layer. It detects known attacks using rules. These are techniques used to define and describe the normal operations for detecting selective forwarding attacks. Rules must be applied before nodes are deployed in a network area. The rule-based processing layer checks the traffic by comparing it to a list of rules. If the traffic satisfies at least 90% of the rules, the node is confirmed to be legitimate (Algorithm 2). Therefore, the traffic will be returned to the pool of MAC IDs for release. If the traffic does not satisfy 90% of the rules, the node is considered doubtful and is rejected. Details of the rules are given in Table 1.

**Algorithm 2.** Rules Processing Layer

1.   Input = (RP: Rules Process)
2.   Output = (DT: Selective Forwarding Detector, RU: Rules)
3.   Network parameter = (SN: Sensor node, RT: Route)
4.   Attacking parameter = (SFAT: Attacker)
5.   RL1 = Rules based in IDS (RL1IDS)
6.   RP ⊆ RL1IDS
7.      Set RL1 >= RU // 90% from the rules
8.   For (SFAT = RL1; SFAT <= RP; SFAT ++)
9.    If SFAT ⊆ RP then
10.      DT → SFAT
11.      Attack alert
12.      Rejected
13.      Dropped
14.    Else if (SFAT ⊄ RP) then
15.      Set SN = RT
16.      SN → AD
17.    End if
18.    End else
19.   End for

**Table 1:** Rules based in selective forwarding attack.

Rule No.	Rule Description
Rule1	Each node wait to see if the neighbor node forward the message or not.
Rule2	The node that will receives message has to checks the transfer’s identity to make sure it is not change during transferring.
Rule3	Each node makes sure that the next node has a shared key for negotiation.
Rule4	Each node has a message route when it wants to transfer to other node.
Rule5	Each sensor node must have ACKs.
Rule6	Each sensor node must have the same ACK that use.
Rule7	Each node has not created a new response before the previous one transfer.
Rule8	Each node has to send the message using the correct route.
Rule9	Each sensor node only communicates with other sensor nodes that locate in the same topology.

#### 4.3.3. Anomaly Detection Layer Based on Intrusion Detection System

The third layer involves anomaly detection, which is the recognition of unknown attacks. This layer checks the traffic that comes from the rule-based processing layer. Therefore, it works to analyze the traffic. The possible results of anomaly detection are false negative, false positive, true negative, and true positive. If the algorithm determines that an unknown attack is a false negative, it sends an alert and rejects the relevant packet. Otherwise, the traffic is returned to the pool of MAC IDs by confirming the legitimacy of the node as shown in Algorithm 3.

**Algorithm 3.** Anomaly Detection Layer Based on IDS

1.   Input = (AD: Anomaly Detection)
2.   Output = (DT: Selective Forwarding Detector)
3.   Network parameter = (SN: Sensor node, RT: Route)
4.   Attacking parameter = (SFAT: Attacker)
5.   RL2 = Anomaly detection based in IDS (RL2IDS)
6.   AD ⊆ RL2IDS
7.   For (RL2 = 0; RL2 <= AD; RL2 ++)
8.      RL2 = RL2 + 1
9.     If RL2 ∈ AD then
10.      Compute FN
11.      FN = 1/N ∑ FN
12.       M = 1
13.      Set Alert
14.      Rejected
15.      Dropped
16.     Else if RL2 ∉ AD then
17.      No Attack
18.      Set SN = RT
19.      Return
20.      SN → MP
21.      Declared
22.     End if
23.     End else
24.   End for

## 5. Reliable, Energy Efficient and Scalable (RES) Model

The goal of a reliable, energy-efficient and scalable (RES) model is to extend the network lifetime while maintaining the Quality of Service (QoS). The network lifetime is the most significant metric of wireless sensor networks. RES also aims to balance the energy utilization for unevenly distributed sensor nodes to provide longer secure surveillance for military bases. In the military base surveillance, there is a high probability that nodes will die by forwarding heavy traffic.

In order to develop reliable communication, we have to determine a reliable path from the sender node to the base station, as the ∀K number of the sensor nodes in the reliable optimal RP path is given as:

(1) $$\overset{\forall K}{\underset{i=0}{\prod }}RP_{ij}$$

Let us assume that WSNs are perceived as the 2D graph with vertex V and edges E written as G(V,E) with transmission range $T_{r}$ so that the maximum reliable communication can be obtained using Bellman-Ford algorithm’s link measurement properties BF given as:

(2) $$BF=\frac{\theta {\sigma_{y}}^{2}}{T_{r}{d_{x}}^{-n}}$$

Once, we start searching the reliable path for communication then we can this write as:

(3) $$RP\;:RP_{max}\;\overset{N}{\underset{\left(i,j\right)}{\prod }}RP_{ij}\;-\;RP_{min}\;\overset{N}{\underset{\left(i,j\in RP\right)}{\sum }}-log\frac{1}{RP_{ij}}$$

Once we are able to find the reliable communication pathway, then we have to balance the energy consumption. We define the network lifetime when the sensor node first time drains its energy. Ideally, prolonging the network lifetime requires satisfying the following conditions: total consumed energy for all sensor nodes in the network, the differences between the node’s individual energy consumption, average energy consumption of each sensor node, and energy consumed for transmitting the packet and for receiving the packet.

Total consumed energy for all sensor nodes in the network should be considered as minimal $\prod^{\text{​}}∆E_{m}$. Determining the differences between the node’s individual energy consumption $∆E_{m}(1\leq k\;\leq \;S_{n}$) and an average energy consumption $∆E_{a}$ is the minimal energy. The differences can be accumulated as:

(4) $$\rho^{2}=\overset{n}{\underset{k=0}{\sum }}k{\left(∆E_{m}-\;∆E_{a}\right)}^{2}$$

where $\rho^{2}$ is differences between minimal energy and an average energy of the sensor node.

After determining the differences, we focuses on an average energy $∆E_{a}$ consumption of each sensor node that can be written as:

(5) $$∆E_{a}=\;\overset{n}{\underset{k=0}{\sum }}k\left(∆E_{m}\right)$$

As well as, we need to determine the number of generated packets generated by sensor node k:

(6) $$\omega_{p}=\;\left(∆\beta_{t}\;\overset{n}{\underset{u\in S\left(k\right)}{\prod }}Y_{uk}-∆\gamma_{r}\;\overset{n}{\underset{v\;\in \;S\left(k\right)}{\prod }}Z_{vk}\;\right)$$

Once a node joins and leaves the network, the communication performance is affected and the provision of QoS is degraded. We address scalability in our design to overcome the performance degradation.

Let us consider the number of joining nodes $k_{j}\;$ in the network. The size of the network is limited and it does not accept a load of more than $k_{j}\leq 1\;\leq \;k_{t}\;$. Given that the network will accept $k_{l}$ sensor nodes in the network, thus, scalable probability of network can be defined as:

(7) $${S_{p}}^{+}=\overset{\propto }{\underset{k=0}{\sum }}(k_{t})+k_{j}\times \;\iint_{i=0\;\&\;j=0}^{N+}{\left(∆p\right)}^{n}\;+\left(\nabla \text{p}\right)$$

where ${\left(∆p\right)}^{n}$ the number of delivered packets from the sensor nodes that are already part of the network and ∇p the number of packets delivered by nodes joining the network and ${S_{p}}^{+}$ the scalable probability when sensor nodes join the networks.

## 6. Results and Discussion

**Figure 4:**
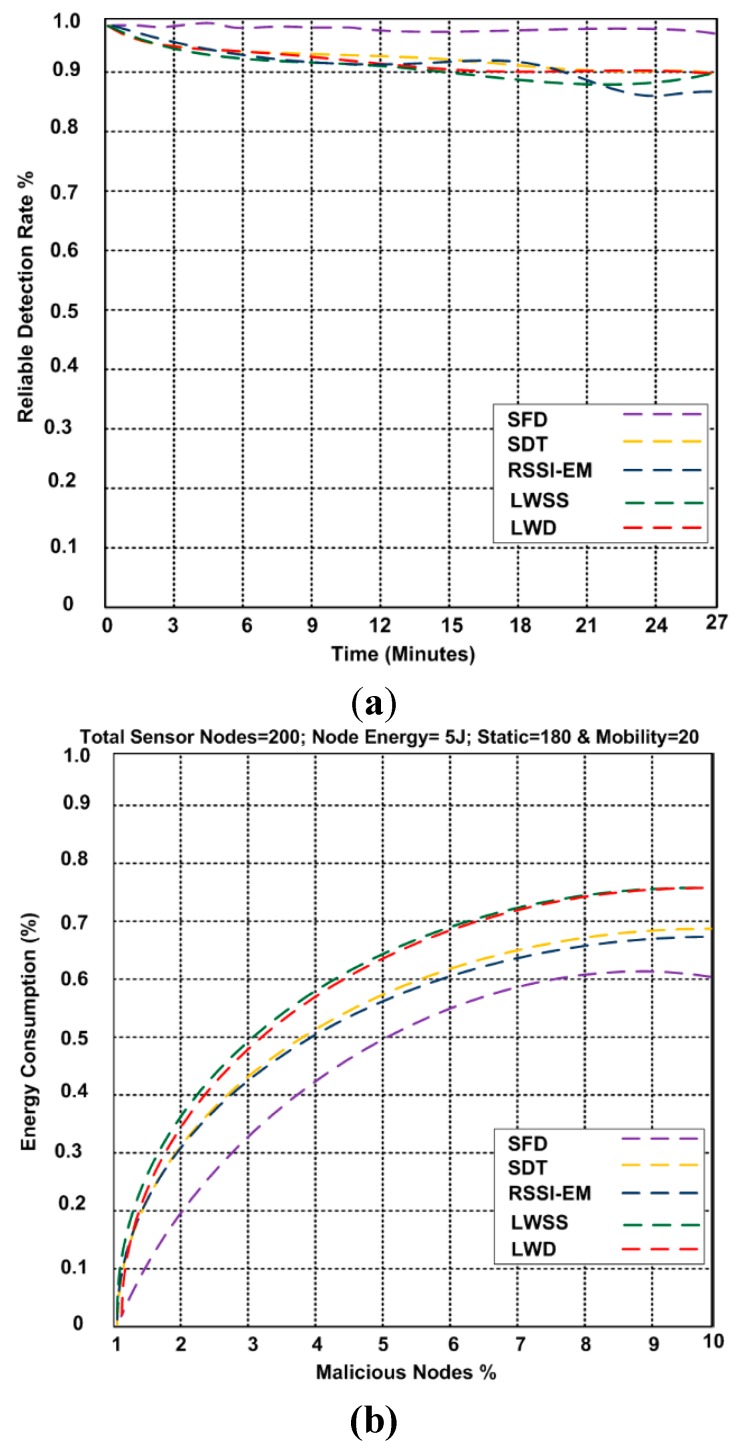

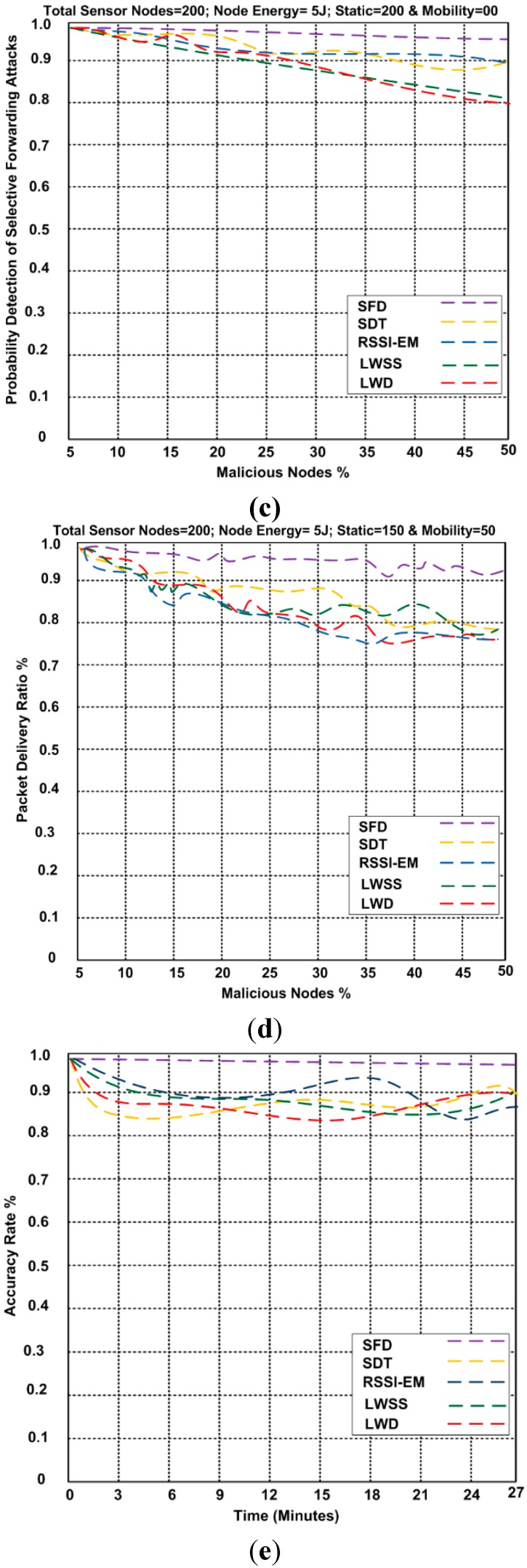
(**a**) Reliable detection rate of selective forwarding attack; (**b**) Energy consumptions; (**c**) Probability detection of selective forwarding attack; (**d**) Packet delivery ratio; (**e**) Accuracy rate.

The approach to detecting selective forwarding attacks is tested using a simulation. In the simulation, 200 sensor nodes are deployed in a network with an area of 800 × 800 m^2^ using NS2 (Table 2). Therefore, each node had a transmission range of 35 m and a sensing range of 30 m. The energetic cost of a node is 5 J, and there are 180 static and 20 mobile nodes. We calculated the amount of energy consumed. Figure 4a describes the reliable detection rate of our approach and other works. The reliable detection rate is important to extend the network lifetime. We proved the number of packets successfully received at the destination node. It clearly shows that SFD is stable at almost the same level when the time increased from 0 min to 27 min. The reliable detection rate is 98.4%. The reliable detection rate for the LWSS, LWD, SDT, and RSSI-EM approaches are not stable and go down when the time increases. The reliability rates are 88.2%, 90.6%, 89.6%, and 86.3%, respectively. Energy is also an important factor. Figure 4b shows the energy consumption performance of the LWSS, LWD, SDT, and RSSI-EM approaches with 180 static nodes and 20 mobility nodes. In comparing our proposed SFD approach with the other approaches, we assume the 10% of nodes are malicious and 10% of the nodes are mobile. As a result, we saw different percentages of energy consumption for each one of these approaches, which consumed 75.1%, 81.8%, 69.1%, and 68.5%, respectively. Thus, the total of malicious nodes and energy consumption appears. Figure 4c shows the probability detection of selective forwarding attacks and other competing schemes with 50% malicious nodes and static nodes. As a result, SFD has a high probability of almost 96%. In Figure 4d we show the packet delivery ratio with 50% malicious nodes and 25% mobile nodes. Between 5% and 10% malicious nodes, the SFD approach has a ratio of 99.2%, higher than the values of the other approaches which 94.4%, 94.1%, 94.3%, and 94.2%. The accuracy rate of SFD and other competing selective forwarding mechanisms are shown in Figure 4e. The accuracy of our approach is more than 98%. The network consumes less energy when it includes mobile nodes; therefore, it was 60.4% at the highest point, and the energy cost was low. If there are malicious nodes along the routes, the SFD approach is able to reduce the communication overhead. The new approach is more effective while the detection of nodes is increased.

**Table 2:** Experiment Parameters.

Parameters	Description
Transmission Range	35 m
Sensing Range of node	30 m
Initial energy of a node	5 J
Bandwidth of node	60 Kb/Sec
Number of legitimate sensors	120
Number of Malicious nodes	80
Size of network	800 × 800 m^2^
Buffering capacity	45 Packets buffering capacity at each node
Data Packet size	128 bytes
Simulation time	27 min
Tx energy	15.2 mW
Rx energy	11.8 mW
Power Intensity	−18 dBm to 13 dBm.

## 7. Conclusions

Security, reliability, energy efficiency, and scalability are challenging design issues for wireless sensor networks. We present in this work a new approach, called Selective Forwarding Detection (SFD), to detect one type of severe attack, selective forwarding attacks. This type of attack severaly affects the communication network of nodes by breaking the communication links. It is a multi-layer detection approach. The multi-layer detection framework consists of three layers, each of which is supported by a different algorithm. In the first layer, we used an algorithm based on a pool of MAC IDs that authenticates the incoming traffic to determine whether a node is legitimate or malicious. In the second layer, we used a rule-based processing algorithm, which checks the traffic by comparing it to a list of rules. In the third layer, we used an anomaly detection algorithm to identify unknown attacks, which appear as false negatives, send an alert, and reject the traffic. In addition, the framework was validated using NS2. Based on the simulation results, we demonstrated that this approach’s detection rate and energy consumption are better than other approaches, therefore, the FD approach is a reliable, energy efficient, and scalable technique to prevent forwarding attacks.
